# *N*-Methyl-3,5-dinitro­benzamide

**DOI:** 10.1107/S1600536811053888

**Published:** 2011-12-21

**Authors:** Gui-Ming Deng, Chao-Run Wang, Zhen Chen, He-Ming Zhang

**Affiliations:** a MOE Key Laboratory of Laser Life Science & Institute of Laser Life Science, College of Biophotonics, South China Normal University, Guangzhou 510631, People’s Republic of China; b The First Affiliated Hospital of Hunan University of Chinese Medicine, Changsha 410007, People’s Republic of China

## Abstract

The asymmetric unit of the title compound, C_8_H_7_N_3_O_5_, contains two independent mol­ecules in which the amide plane is oriented at dihedral angles of 29.82 (2) and 31.17 (2)° with respect to the benzene ring. In the crystal, mol­ecules are connected *via* inter­molecular N—H⋯O hydrogen bonds, forming chains running along the *b* axis.

## Related literature

For general background to the biological activity of benzamide derivatives, see: Lee *et al.* (2009 ▶). For bond-length data, see: Allen *et al.* (1987 ▶).
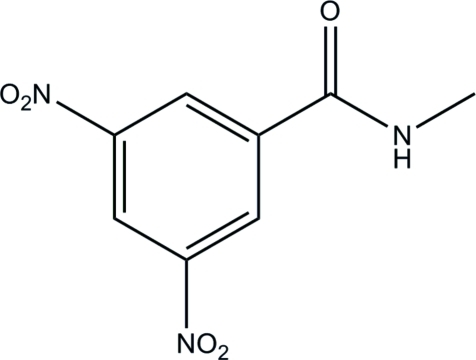


## Experimental

### 

#### Crystal data

C_8_H_7_N_3_O_5_
*M* *_r_* = 225.16
Orthorhombic, 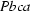

*a* = 10.716 (2) Å
*b* = 10.057 (2) Å
*c* = 36.101 (7) Å
*V* = 3890.6 (13) Å^3^
*Z* = 16
Mo *K*α radiation
μ = 0.13 mm^−1^
*T* = 293 K
0.30 × 0.20 × 0.10 mm

#### Data collection

Enraf–Nonius CAD-4 diffractometer
Absorption correction: ψ scan (North *et al.*, 1968 ▶) *T*_min_ = 0.962, *T*_max_ = 0.987
4711 measured reflections
3402 independent reflections
1653 reflections with *I* > 2σ(*I*)
*R*_int_ = 0.0582
3 standard reflections every 200 reflections  intensity decay: 1%

#### Refinement

*R*[*F* ^2^ > 2σ(*F* ^2^)] = 0.067
*wR*(*F* ^2^) = 0.162
*S* = 0.96
3402 reflections
289 parameters
H-atom parameters constrained
Δρ_max_ = 0.21 e Å^−3^
Δρ_min_ = −0.17 e Å^−3^

### 

Data collection: *CAD-4 Software* (Enraf–Nonius, 1985 ▶); cell refinement: *CAD-4 Software*; data reduction: *XCAD4* (Harms & Wocadlo, 1995 ▶); program(s) used to solve structure: *SHELXTL* (Sheldrick, 2008 ▶); program(s) used to refine structure: *SHELXTL*; molecular graphics: *SHELXTL*; software used to prepare material for publication: *SHELXTL*.

## Supplementary Material

Crystal structure: contains datablock(s) I, global. DOI: 10.1107/S1600536811053888/xu5409sup1.cif

Structure factors: contains datablock(s) I. DOI: 10.1107/S1600536811053888/xu5409Isup2.hkl

Supplementary material file. DOI: 10.1107/S1600536811053888/xu5409Isup3.cml

Additional supplementary materials:  crystallographic information; 3D view; checkCIF report

## Figures and Tables

**Table 1:** Hydrogen-bond geometry (Å, °)

*D*—H⋯*A*	*D*—H	H⋯*A*	*D*⋯*A*	*D*—H⋯*A*
N3—H3*A*⋯O5^i^	0.86	2.06	2.900 (4)	165
N6—H6*A*⋯O10^i^	0.86	2.12	2.955 (4)	164

Symmetry code: (i) 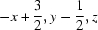.

**Fig. 1.**
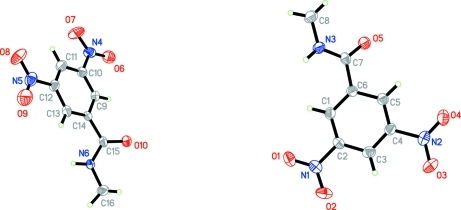
The molecular structure of (I) (thermal ellipsoids are shown at 30% probability levels).

**Fig. 2.**
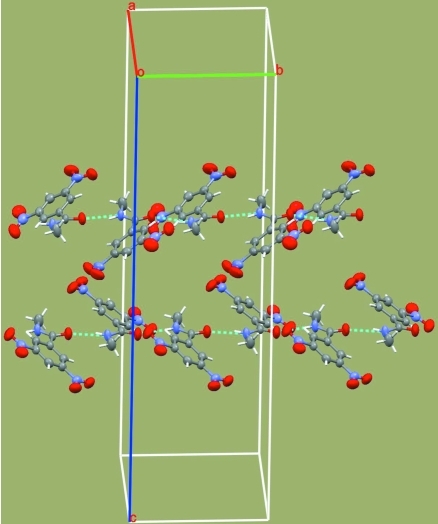
The packing of (I), viewed down the b axis. The dashed lines represent the hydrogen bonding interactions.

## supplementary crystallographic information

### Comment

Benzamide derivatives exhibit interesting biological activities such as antibacterial and antifungal effects (Lee *et al.*., 2009). We report here the crystal structure of the title compound (Fig. 1). Bond lengths and angles are within normal ranges (Allen *et al.*, 1987).

In the crystal packing of (I) the molecules are connected together *via* N—H···O intermolecular hydrogen bonds to form a one-dimensional chains in the *b* direction (Table 1, graph set C1,1(4)), which seems to be very effective in the stabilization of the crystal structure.

### Experimental

A 1:1 mixture of N,N'-dimethylurea (0.088 g, 1 mmol), 3,5-dinitrobenzoic acid (0.212 g, 1 mmol) and ZrOCl_2_.8H_2_O (0.032 g, 1 mmol) were ground in a mortar, placed in a 50 ml conical flask and irradiated in a microwave oven. The progress of the reaction was monitored by TLC. After completion of the reaction, the contents were extracted with EtOAc (3 × 10 ml) and filtered to remove the catalyst. To remove unreacted acid, the organic layer was washed with a NaHCO_3_ solution followed by water. Evaporating the organic solvent provided the crystals.

### Refinement

All H atoms were positioned geometrically and constrained to ride on their parent atoms, with C—H = 0.93 Å for aromatic H, 0.96 Å for methyl H and 0.86 Å for N—H. U_iso_(H)= 1.2U_eq_(C) and 1.5U_eq_(N).

### Crystal data

**Table:**

C_8_H_7_N_3_O_5_	*F*(000) = 1856
*M**_r_* = 225.16	*D*_x_ = 1.538 Mg m^−^^3^
Orthorhombic, *P**b**c**a*	Mo *K*α radiation, λ = 0.71073 Å
Hall symbol: -P 2ac 2ab	Cell parameters from 25 reflections
*a* = 10.716 (2) Å	θ = 9–13°
*b* = 10.057 (2) Å	µ = 0.13 mm^−^^1^
*c* = 36.101 (7) Å	*T* = 293 K
*V* = 3890.6 (13) Å^3^	Block, yellow
*Z* = 16	0.30 × 0.20 × 0.10 mm

### Data collection

**Table:**

Enraf–Nonius CAD-4 diffractometer	1653 reflections with *I* > 2σ(*I*)
Radiation source: fine-focus sealed tube	*R*_int_ = 0.058
graphite	θ_max_ = 25.1°, θ_min_ = 2.2°
ω/2θ scans	*h* = 0→12
Absorption correction: ψ scan (North *et al.*, 1968)	*k* = 0→11
*T*_min_ = 0.962, *T*_max_ = 0.987	*l* = 0→42
4711 measured reflections	3 standard reflections every 200 reflections
3402 independent reflections	intensity decay: 1%

### Refinement

**Table:**

Refinement on *F*^2^	Primary atom site location: structure-invariant direct methods
Least-squares matrix: full	Secondary atom site location: difference Fourier map
*R*[*F*^2^ > 2σ(*F*^2^)] = 0.067	Hydrogen site location: inferred from neighbouring sites
*wR*(*F*^2^) = 0.162	H-atom parameters constrained
*S* = 0.96	*w* = 1/[σ^2^(*F*_o_^2^) + (0.0619*P*)^2^] where *P* = (*F*_o_^2^ + 2*F*_c_^2^)/3
3402 reflections	(Δ/σ)_max_ < 0.001
289 parameters	Δρ_max_ = 0.21 e Å^−^^3^
0 restraints	Δρ_min_ = −0.17 e Å^−^^3^

### Special details

**Table:**

Geometry. All e.s.d.'s (except the e.s.d. in the dihedral angle between two l.s. planes) are estimated using the full covariance matrix. The cell e.s.d.'s are taken into account individually in the estimation of e.s.d.'s in distances, angles and torsion angles; correlations between e.s.d.'s in cell parameters are only used when they are defined by crystal symmetry. An approximate (isotropic) treatment of cell e.s.d.'s is used for estimating e.s.d.'s involving l.s. planes.
Refinement. Refinement of *F*^2^ against ALL reflections. The weighted *R*-factor *wR* and goodness of fit *S* are based on *F*^2^, conventional *R*-factors *R* are based on *F*, with *F* set to zero for negative *F*^2^. The threshold expression of *F*^2^ > σ(*F*^2^) is used only for calculating *R*-factors(gt) *etc*. and is not relevant to the choice of reflections for refinement. *R*-factors based on *F*^2^ are statistically about twice as large as those based on *F*, and *R*- factors based on ALL data will be even larger.

### Fractional atomic coordinates and isotropic or equivalent isotropic displacement parameters (Å^2^)

**Table:**

	*x*	*y*	*z*	*U*_iso_*/*U*_eq_	
N1	1.1588 (3)	0.7222 (4)	0.65015 (10)	0.0554 (9)	
C1	0.9707 (3)	0.8426 (4)	0.67035 (11)	0.0444 (10)	
H1A	0.9221	0.7795	0.6582	0.053*	
O1	1.0927 (3)	0.6404 (3)	0.63547 (10)	0.0798 (11)	
O2	1.2734 (2)	0.7187 (3)	0.64960 (8)	0.0716 (9)	
C2	1.0989 (3)	0.8326 (4)	0.67030 (11)	0.0462 (10)	
N2	1.1949 (4)	1.1247 (4)	0.72609 (10)	0.0571 (10)	
N3	0.7009 (3)	0.8718 (3)	0.68582 (10)	0.0568 (10)	
H3A	0.7314	0.7927	0.6850	0.068*	
O3	1.3077 (3)	1.1073 (3)	0.72618 (9)	0.0726 (10)	
C3	1.1751 (3)	0.9227 (4)	0.68860 (12)	0.0521 (11)	
H3B	1.2616	0.9148	0.6885	0.063*	
O4	1.1423 (3)	1.2198 (3)	0.74015 (10)	0.0745 (10)	
C4	1.1155 (3)	1.0243 (4)	0.70688 (12)	0.0461 (10)	
O5	0.7395 (2)	1.0907 (2)	0.68914 (9)	0.0636 (9)	
C5	0.9889 (3)	1.0400 (4)	0.70711 (11)	0.0437 (10)	
H5A	0.9527	1.1114	0.7194	0.052*	
C6	0.9151 (3)	0.9481 (3)	0.68874 (10)	0.0413 (10)	
C7	0.7776 (3)	0.9749 (4)	0.68801 (11)	0.0445 (10)	
C8	0.5670 (3)	0.8895 (4)	0.68472 (14)	0.0705 (14)	
H8A	0.5272	0.8042	0.6832	0.106*	
H8B	0.5450	0.9416	0.6634	0.106*	
H8C	0.5401	0.9344	0.7068	0.106*	
O6	0.3317 (3)	0.2716 (3)	0.39302 (10)	0.0788 (11)	
O7	0.1844 (3)	0.1345 (4)	0.40663 (10)	0.0918 (12)	
O8	0.2726 (3)	−0.2445 (4)	0.48397 (10)	0.0938 (12)	
O9	0.4620 (4)	−0.2891 (4)	0.49911 (11)	0.0952 (12)	
O10	0.7598 (2)	0.1750 (2)	0.42863 (8)	0.0614 (9)	
N4	0.2939 (3)	0.1676 (4)	0.40662 (11)	0.0617 (11)	
N5	0.3840 (4)	−0.2228 (4)	0.48302 (11)	0.0669 (11)	
N6	0.8109 (3)	−0.0414 (3)	0.43239 (9)	0.0503 (10)	
H6A	0.7843	−0.1212	0.4356	0.060*	
C9	0.5097 (3)	0.1061 (4)	0.42133 (11)	0.0452 (10)	
H9A	0.5364	0.1806	0.4083	0.054*	
C10	0.3841 (3)	0.0788 (4)	0.42467 (12)	0.0501 (11)	
C11	0.3408 (4)	−0.0288 (4)	0.44465 (12)	0.0560 (11)	
H11A	0.2558	−0.0460	0.4469	0.067*	
C12	0.4279 (3)	−0.1087 (4)	0.46088 (11)	0.0481 (10)	
C13	0.5549 (3)	−0.0875 (3)	0.45744 (11)	0.0468 (10)	
H13A	0.6116	−0.1457	0.4683	0.056*	
C14	0.5960 (3)	0.0213 (3)	0.43772 (11)	0.0390 (9)	
C15	0.7307 (3)	0.0574 (3)	0.43282 (11)	0.0435 (10)	
C16	0.9431 (3)	−0.0190 (4)	0.42662 (15)	0.0718 (14)	
H16A	0.9863	−0.1025	0.4270	0.108*	
H16B	0.9748	0.0372	0.4460	0.108*	
H16C	0.9557	0.0234	0.4031	0.108*	

### Atomic displacement parameters (Å^2^)

**Table:**

	*U*^11^	*U*^22^	*U*^33^	*U*^12^	*U*^13^	*U*^23^
N1	0.063 (2)	0.052 (2)	0.051 (3)	0.010 (2)	0.003 (2)	−0.001 (2)
C1	0.046 (2)	0.039 (2)	0.048 (3)	−0.0017 (18)	−0.004 (2)	−0.002 (2)
O1	0.082 (2)	0.064 (2)	0.093 (3)	−0.0010 (18)	0.012 (2)	−0.035 (2)
O2	0.0549 (19)	0.091 (2)	0.069 (2)	0.0238 (17)	0.0071 (17)	−0.0031 (19)
C2	0.052 (2)	0.038 (2)	0.049 (3)	0.0070 (19)	0.003 (2)	−0.001 (2)
N2	0.058 (2)	0.057 (2)	0.056 (3)	−0.012 (2)	−0.010 (2)	0.000 (2)
N3	0.0419 (17)	0.0308 (16)	0.098 (3)	−0.0009 (15)	−0.0010 (18)	0.007 (2)
O3	0.0486 (17)	0.091 (2)	0.078 (2)	−0.0116 (17)	−0.0149 (17)	0.003 (2)
C3	0.043 (2)	0.052 (2)	0.061 (3)	0.005 (2)	−0.003 (2)	0.009 (2)
O4	0.077 (2)	0.0621 (19)	0.085 (3)	−0.0036 (18)	−0.0174 (18)	−0.021 (2)
C4	0.042 (2)	0.042 (2)	0.055 (3)	−0.0048 (19)	−0.004 (2)	0.002 (2)
O5	0.0520 (16)	0.0334 (15)	0.106 (3)	0.0035 (13)	−0.0022 (17)	−0.0014 (15)
C5	0.048 (2)	0.040 (2)	0.043 (2)	−0.0008 (19)	0.0021 (19)	0.001 (2)
C6	0.042 (2)	0.034 (2)	0.048 (3)	0.0019 (18)	0.000 (2)	0.004 (2)
C7	0.054 (2)	0.031 (2)	0.049 (3)	0.001 (2)	0.001 (2)	0.001 (2)
C8	0.045 (2)	0.051 (2)	0.116 (4)	−0.001 (2)	0.000 (3)	0.002 (3)
O6	0.068 (2)	0.066 (2)	0.102 (3)	0.0145 (19)	−0.019 (2)	0.010 (2)
O7	0.0410 (17)	0.129 (3)	0.106 (3)	−0.0026 (19)	−0.0210 (18)	0.015 (2)
O8	0.072 (2)	0.117 (3)	0.092 (3)	−0.041 (2)	0.016 (2)	0.019 (2)
O9	0.099 (3)	0.079 (3)	0.108 (3)	−0.009 (2)	0.016 (2)	0.032 (2)
O10	0.0528 (16)	0.0324 (14)	0.099 (2)	−0.0012 (13)	0.0006 (17)	0.0076 (16)
N4	0.046 (2)	0.075 (3)	0.064 (3)	0.010 (2)	−0.016 (2)	−0.014 (2)
N5	0.073 (3)	0.069 (3)	0.058 (3)	−0.020 (2)	0.014 (2)	0.000 (2)
N6	0.0394 (17)	0.0264 (16)	0.085 (3)	−0.0011 (14)	0.0063 (17)	0.0019 (18)
C9	0.046 (2)	0.035 (2)	0.055 (3)	−0.0028 (18)	0.000 (2)	−0.003 (2)
C10	0.046 (2)	0.049 (2)	0.056 (3)	0.003 (2)	−0.008 (2)	−0.014 (2)
C11	0.051 (2)	0.061 (3)	0.055 (3)	−0.007 (2)	0.005 (2)	−0.009 (2)
C12	0.053 (2)	0.044 (2)	0.047 (3)	−0.010 (2)	0.009 (2)	0.001 (2)
C13	0.055 (2)	0.037 (2)	0.049 (3)	−0.0001 (19)	0.002 (2)	−0.004 (2)
C14	0.040 (2)	0.0296 (18)	0.047 (2)	0.0027 (17)	−0.0007 (18)	−0.0053 (19)
C15	0.048 (2)	0.027 (2)	0.056 (3)	−0.0007 (18)	−0.003 (2)	−0.0014 (19)
C16	0.045 (2)	0.049 (2)	0.121 (4)	−0.002 (2)	0.007 (3)	0.016 (3)

### Geometric parameters (Å, °)

**Table:**

N1—O1	1.207 (4)	O6—N4	1.224 (4)
N1—O2	1.229 (4)	O7—N4	1.221 (4)
N1—C2	1.475 (5)	O8—N5	1.214 (4)
C1—C2	1.378 (5)	O9—N5	1.217 (4)
C1—C6	1.386 (5)	O10—C15	1.232 (4)
C1—H1A	0.9300	N4—C10	1.468 (5)
C2—C3	1.387 (5)	N5—C12	1.475 (5)
N2—O4	1.221 (4)	N6—C15	1.314 (4)
N2—O3	1.221 (4)	N6—C16	1.449 (4)
N2—C4	1.491 (5)	N6—H6A	0.8600
N3—C7	1.325 (4)	C9—C10	1.379 (5)
N3—C8	1.446 (4)	C9—C14	1.391 (5)
N3—H3A	0.8600	C9—H9A	0.9300
C3—C4	1.374 (5)	C10—C11	1.381 (5)
C3—H3B	0.9300	C11—C12	1.364 (5)
C4—C5	1.366 (5)	C11—H11A	0.9300
O5—C7	1.234 (4)	C12—C13	1.383 (5)
C5—C6	1.385 (5)	C13—C14	1.378 (5)
C5—H5A	0.9300	C13—H13A	0.9300
C6—C7	1.498 (5)	C14—C15	1.498 (5)
C8—H8A	0.9600	C16—H16A	0.9600
C8—H8B	0.9600	C16—H16B	0.9600
C8—H8C	0.9600	C16—H16C	0.9600
			
O1—N1—O2	124.0 (4)	O7—N4—O6	123.5 (4)
O1—N1—C2	118.3 (3)	O7—N4—C10	117.8 (4)
O2—N1—C2	117.6 (4)	O6—N4—C10	118.7 (3)
C2—C1—C6	119.0 (3)	O8—N5—O9	124.3 (4)
C2—C1—H1A	120.5	O8—N5—C12	118.0 (4)
C6—C1—H1A	120.5	O9—N5—C12	117.7 (4)
C1—C2—C3	122.6 (4)	C15—N6—C16	121.6 (3)
C1—C2—N1	119.3 (4)	C15—N6—H6A	119.2
C3—C2—N1	118.1 (3)	C16—N6—H6A	119.2
O4—N2—O3	124.6 (4)	C10—C9—C14	119.4 (4)
O4—N2—C4	117.4 (3)	C10—C9—H9A	120.3
O3—N2—C4	118.0 (4)	C14—C9—H9A	120.3
C7—N3—C8	121.3 (3)	C9—C10—C11	122.0 (4)
C7—N3—H3A	119.3	C9—C10—N4	118.8 (4)
C8—N3—H3A	119.3	C11—C10—N4	119.2 (4)
C4—C3—C2	116.2 (3)	C12—C11—C10	117.1 (4)
C4—C3—H3B	121.9	C12—C11—H11A	121.4
C2—C3—H3B	121.9	C10—C11—H11A	121.4
C5—C4—C3	123.4 (4)	C11—C12—C13	122.9 (4)
C5—C4—N2	119.1 (4)	C11—C12—N5	118.2 (4)
C3—C4—N2	117.5 (3)	C13—C12—N5	118.9 (4)
C4—C5—C6	119.2 (4)	C14—C13—C12	119.0 (4)
C4—C5—H5A	120.4	C14—C13—H13A	120.5
C6—C5—H5A	120.4	C12—C13—H13A	120.5
C5—C6—C1	119.6 (3)	C13—C14—C9	119.6 (3)
C5—C6—C7	116.7 (3)	C13—C14—C15	124.2 (3)
C1—C6—C7	123.5 (3)	C9—C14—C15	116.2 (3)
O5—C7—N3	122.4 (3)	O10—C15—N6	123.9 (3)
O5—C7—C6	119.6 (3)	O10—C15—C14	119.4 (3)
N3—C7—C6	118.0 (3)	N6—C15—C14	116.6 (3)
N3—C8—H8A	109.5	N6—C16—H16A	109.5
N3—C8—H8B	109.5	N6—C16—H16B	109.5
H8A—C8—H8B	109.5	H16A—C16—H16B	109.5
N3—C8—H8C	109.5	N6—C16—H16C	109.5
H8A—C8—H8C	109.5	H16A—C16—H16C	109.5
H8B—C8—H8C	109.5	H16B—C16—H16C	109.5
			
C6—C1—C2—C3	1.3 (6)	C14—C9—C10—C11	1.7 (6)
C6—C1—C2—N1	−178.8 (3)	C14—C9—C10—N4	−178.8 (3)
O1—N1—C2—C1	−2.9 (6)	O7—N4—C10—C9	172.6 (4)
O2—N1—C2—C1	177.6 (4)	O6—N4—C10—C9	−8.7 (6)
O1—N1—C2—C3	177.0 (4)	O7—N4—C10—C11	−7.8 (6)
O2—N1—C2—C3	−2.5 (5)	O6—N4—C10—C11	170.8 (4)
C1—C2—C3—C4	−0.4 (6)	C9—C10—C11—C12	−0.6 (6)
N1—C2—C3—C4	179.7 (3)	N4—C10—C11—C12	179.9 (4)
C2—C3—C4—C5	−1.0 (6)	C10—C11—C12—C13	−1.3 (6)
C2—C3—C4—N2	−178.6 (3)	C10—C11—C12—N5	178.8 (4)
O4—N2—C4—C5	−2.6 (6)	O8—N5—C12—C11	4.2 (6)
O3—N2—C4—C5	178.6 (4)	O9—N5—C12—C11	−175.9 (4)
O4—N2—C4—C3	175.1 (4)	O8—N5—C12—C13	−175.8 (4)
O3—N2—C4—C3	−3.8 (6)	O9—N5—C12—C13	4.1 (6)
C3—C4—C5—C6	1.4 (6)	C11—C12—C13—C14	2.0 (6)
N2—C4—C5—C6	178.9 (3)	N5—C12—C13—C14	−178.1 (3)
C4—C5—C6—C1	−0.3 (5)	C12—C13—C14—C9	−0.8 (5)
C4—C5—C6—C7	−176.4 (3)	C12—C13—C14—C15	178.3 (4)
C2—C1—C6—C5	−1.0 (5)	C10—C9—C14—C13	−0.9 (6)
C2—C1—C6—C7	174.8 (4)	C10—C9—C14—C15	179.9 (3)
C8—N3—C7—O5	−0.2 (7)	C16—N6—C15—O10	−0.3 (7)
C8—N3—C7—C6	−179.6 (4)	C16—N6—C15—C14	177.9 (3)
C5—C6—C7—O5	29.1 (5)	C13—C14—C15—O10	−151.0 (4)
C1—C6—C7—O5	−146.9 (4)	C9—C14—C15—O10	28.1 (5)
C5—C6—C7—N3	−151.5 (4)	C13—C14—C15—N6	30.6 (6)
C1—C6—C7—N3	32.5 (6)	C9—C14—C15—N6	−150.3 (4)

### Hydrogen-bond geometry (Å, °)

**Table:**

*D*—H···*A*	*D*—H	H···*A*	*D*···*A*	*D*—H···*A*
N3—H3A···O5^i^	0.86	2.06	2.900 (4)	165.
N6—H6A···O10^i^	0.86	2.12	2.955 (4)	164.

Symmetry codes: (i) −*x*+3/2, *y*−1/2, *z*.
